# *Carex* Beyond Taxonomy: Integrating Genomic Architecture, Life History, and Ecosystem Function

**DOI:** 10.3390/antiox15070838

**Published:** 2026-07-02

**Authors:** Shuang Xiao, Xueqing Liu, Yanming Wang, Yuesen Yue, Juying Wu, Haifeng Wen, Hui Zhang, Xifeng Fan

**Affiliations:** Institute of Grassland, Flowers and Ecology, Beijing Academy of Agriculture and Forestry Sciences, Beijing 100097, China; xiaoshuang813@outlook.com (S.X.); liuxueqing@baafs.net.cn (X.L.); 202530221112@bua.edu.cn (Y.W.); yueyuesen@baafs.net.cn (Y.Y.); wujuying@baafs.net.cn (J.W.); wenhaifeng@baafs.net.cn (H.W.)

**Keywords:** *Carex*, holocentric chromosomes, genomic architecture, life history strategies, abiotic stress adaptation, ecosystem functions, bioactive compounds

## Abstract

*Carex* is among the most species-rich genera of angiosperms and plays important ecological roles in wetlands, alpine regions, and temperate ecosystems worldwide. However, research on this genus has long been challenged by pronounced phenotypic plasticity, reduced floral morphology, frequent hybridization, and complex chromosomal evolution. Although recent advances in molecular phylogenetics, comparative genomics, reproductive biology, and ecophysiology have substantially expanded the knowledge of *Carex*, these findings remain fragmented across disciplines. Here, we synthesize current evidence on *Carex* taxonomy and phylogeny, genomic and karyotypic evolution, reproductive and life history strategies, abiotic stress responses, ecosystem functions, and bioresource potential within a cross-scale framework. This review emphasizes how genomic architecture, life history variation, and ecophysiological adaptation jointly shape species diversification and ecosystem functioning, while clarifying their implications for habitat restoration and the sustainable use of *Carex* resources. Finally, we identify key priorities for future research, including improved phylogenomic resolution, comparative functional studies, climate-resilience assessment, and germplasm conservation and sustainable use.

## 1. Introduction

*Carex* L. is the largest genus within the family Cyperaceae and ranks among the three most species-rich genera of angiosperms [1]. Characterized by remarkable morphological plasticity and extensive ecological adaptability, the genus has long been served as a focal taxon in botanical and ecological research [2,3]. As a keystone group in fragile habitats, including northern temperate and Arctic wetlands, alpine steppes, and periglacial zones, *Carex* plays an irreplaceable role in maintaining ecosystem stability by virtue of its unique reproductive strategies, robust clonal propagation capabilities, and strong resilience to stress [4]. Furthermore, the genus possesses significant economic value. Many species not only provide high-quality forage but also show great potential as landscaping ground covers due to their early green-up and prolonged green periods [5].

Despite its massive ecological footprint and application potential, the taxonomic delimitation of *Carex* has historically been notoriously difficult [6,7]. Because the genus exhibits complex morphological variation heavily influenced by environmental plasticity, traditional morphology-based taxonomic methods often fail to accurately resolve phylogenetic relationships. However, in response to growing global imperatives surrounding biodiversity conservation and the restoration of degraded ecosystems, *Carex* research has transcended the boundaries of traditional taxonomy. The field is now rapidly expanding into interdisciplinary domains, particularly genomics and physiological ecology, providing novel perspectives for unraveling the molecular and physiological basis of its adaptive mechanisms.

Accordingly, unlike previous reviews that have mainly focused on individual aspects of *Carex*, such as taxonomy, phylogeny, ecology, or resource utilization, the present review adopts a cross-scale and interdisciplinary framework. This review systematically summarizes recent progress in *Carex* taxonomy and phylogeny, genomic and karyotypic evolution, reproductive and life-histroy strategies, stress adaptation mechanisms, ecosystem functions, and bioresource potential. By linking genomic architecture, life history variation, ecophysiological adaptation, and ecosystem functioning, this review aims to provide an integrated perspective on how the evolutionary characteristics of *Carex* shape both species diversification and ecological roles. This synthesis also highlights future research priorities and provides a reference for germplasm conservation, habitat restoration, and the sustainable use of *Carex* resources.

## 2. Global Distribution and Species Diversity

**Figure 1:**
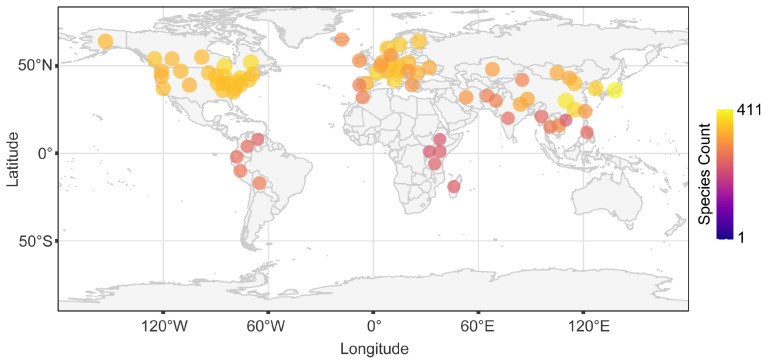
The distribution of the *Carex* genus. Data source: Plants of the World Online (POWO), Royal Botanic Gardens, Kew (https://powo.science.kew.org/results?f=species_f&q=CareX) (accessed on 5 April 2026).

*Carex* ranks among the three largest genera of angiosperms and boasts a nearly cosmopolitan distribution (www.plantsoftheworldonline.org). While taxonomic databases log over 9400 entries for the genus, the currently accepted species count stands at approximately 2000, reflecting its immense diversity and historical taxonomic complexity. Originating in East Asia during the late Eocene, the genus displays a striking inverted latitudinal richness gradient (Figure 1). Consequently, species are relatively rare at low elevations in the tropical Southern Hemisphere and most abundant in the cold and temperate regions of the Northern Hemisphere. Globally, East Asia harbors the highest diversity (~1019 species), followed by the Nearctic (~562 species) [8].

## 3. Taxonomy of *Carex*: Progress and Challenges

*Carex* stands as one of the largest genera of vascular plants, comprising approximately 2000 recognized species worldwide. However, taxonomic research within this genus has historically been considered notoriously challenging [9]. This complexity is primarily attributed to extreme floral reduction, prevalent convergent evolution, frequent interspecific hybridization, and high levels of phenotypic plasticity. Consequently, relying solely on traditional macromorphological characters is often insufficient to clearly delimit species or resolve robust phylogenetic relationships. In recent decades, modern systematics has shifted toward integrating multiple lines of evidence, such as microanatomy, cytogenetics, and molecular biology, to establish a more objective and stable taxonomic basis for this complex group.

### 3.1. Classification Based on Vegetative Organs

Vegetative organs, including leaves, culms, and roots, provide a wealth of taxonomic characters for *Carex*, yet their application requires careful discrimination to avoid interference from environmental plasticity. For instance, within Central European populations of *C. buekii*, soil bioavailable potassium (K^+^) content acts as a key driver of morphological variation. Under low-K^+^ conditions, individuals are generally smaller, and even traditionally critical taxonomic traits, such as utricle and beak lengths, are significantly reduced [10]. Neglecting these habitat effects frequently leads to the erroneous recognition of mere ecotypes as distinct taxonomic units. Similarly, many leaf anatomical characters, including the shape, size, and position of vascular bundles, as well as the quantity and length of bulliform cells, exhibit high intraspecific variation and therefore possess low taxonomic reliability [11]. This highlights that structural qualitative traits are often of greater taxonomic value than quantitative measurements, which are inherently more susceptible to environmental fluctuations.

Nevertheless, vegetative anatomy remains indispensable for delimiting specific taxa. Certain anatomical characters demonstrate high intraspecific consistency, particularly the shape of the transverse leaf section, the relative size of adaxial to abaxial epidermal cells, and the presence of high-density papillae [11]. In morphologically cryptic groups such as *Carex* sect. *Phacocystis*, stomatal distribution patterns (e.g., amphistomous versus hypostomous) and quantitative indices of intercostal cells provide effective means for species delimitation [12]. Furthermore, leaf and culm anatomy serve as powerful tools for distinguishing closely related species, as demonstrated by the successful differentiation of *C. backii* and *C. saximontana* within *Carex* sect. *Phyllostachys* [13]. Belowground, root traits exhibit strong taxonomic signals at the subgeneric level. For instance, dauciform (carrot-shaped) roots appear restricted to the subgenus *Carex*, which also harbors a significantly higher abundance of dark septate endophytes and bulbous (shortened) root hairs compared to the subgenus *Vignea* [14]. This distinction not only separates these taxonomic groups but also reveals profound evolutionary divergence in their belowground nutrient acquisition strategies.

### 3.2. Classification Based on Reproductive Organs

Traditional taxonomy relies heavily on reproductive organs; however, floral reduction and convergent evolution within *Carex* have led to significant controversies regarding macromorphological characters, such as inflorescence structure. Even the terminology for the genus’s most iconic feature, the sac-like structure enveloping the achene, remains inconsistently applied between “perigynium” and “utricle” [15]. These challenges have prompted a shift toward micromorphological characters, particularly facilitated by the application of scanning electron microscopy, revealing a significant “scale-dependency” in their taxonomic utility.

In terms of pollen micromorphology, *Carex* is characterized by a unique “pseudomonad” developmental pattern, where only one of the four microspores develops normally while the remaining three abort [16]. Furthermore, morphological examinations reveal that exine thickness can clearly delimit the subgenera *Vignea* and *Carex*. However, this specific trait possesses almost no taxonomic value at ranks below the subgenus level, such as section or species.

Achene epidermal micromorphology, particularly intracellular silica deposits, exhibits a similar taxonomic pattern. At a macro-scale, these features display high levels of homoplasy. For instance, species with vastly different overall morphologies, traditionally placed in different sections (e.g., within *Carex* sect. *Hymenochlaenae*), can possess nearly indistinguishable achene surface features [17]. Furthermore, the presence of a single conical silica body is likely a plesiomorphy retained across distantly related lineages or a result of parallel evolution, making it an unreliable character for delimiting “sectional” taxonomic units [18]. Conversely, within clearly defined, monophyletic small groups, the diagnostic value of these micromorphological characters becomes highly significant. In *Carex* sect. *Phyllostachys*, for example, achene silica body characteristics are strongly congruent with molecular phylogenetic hypotheses, providing robust support for splitting the *C. willdenowii* complex into three independent species [13]. Even in the absence of broader diagnostic morphological traits, as observed in *C. jamesii*, the intraspecific variation in these silica bodies aligns closely with genetic diversity, demonstrating their effectiveness in reflecting population structure.

### 3.3. Classification Based on Molecular Methods

Holocentric chromosomes represent a core cytogenetic feature of *Carex*. Unlike monocentric chromosomes, in which spindle microtubules attach to a single localized centromere, holocentric chromosomes have kinetochore activity distributed along almost the entire chromosome length. This structure allows chromosome fragments to be transmitted more stably during cell division, thereby facilitating chromosomal fission and fusion that lead to extensive aneuploidy (*n* = 6–68). This mechanism of chromosomal rearrangement has been proven to significantly influence lineage diversification rates and acts as a key driver of speciation and rapid radiation within the genus.

Molecular phylogenetic studies have revealed that the traditional genus *Carex* (s.s.) was, in fact, a paraphyletic group. Supported by robust molecular evidence, the establishment of a monophyletic framework for *Carex* (s.l.) necessitated the incorporation of allied genera, specifically *Kobresia*, *Uncinia*, and *Schoenoxiphium*. Building upon this expanded circumscription, advanced genomic tools, particularly targeted enrichment sequencing (HybSeq), have successfully overcome the lack of phylogenetic signal inherent in traditional markers. This methodological shift has culminated in the proposal of a new, highly resolved classification system comprising six subgenera, including *Carex*, *Vignea*, and *Psyllophora* [19].

However, reticulate evolution remains a major obstacle in *Carex* taxonomy. For example, within the taxonomically challenging *Carex* sect. *Ceratocystis*, severe incongruence between nuclear (ITS) and plastid (rps16, 5′-trnK) gene trees, coupled with numerous additive polymorphisms in ITS sequences, confirms the widespread prevalence of introgression [20]. Consequently, due to rapid lineage radiation and frequent hybridization, the identification success rate of standard DNA barcodes (e.g., matK, rbcL, trnH-psbA) is extremely low, ranging from only 19% to 38% [21]. Beyond recent radiations, this paucity of interspecific genetic variation and poor barcode resolution is heavily driven by the exceptionally slow molecular evolutionary rate of the *Carex* plastid genome [21]. Therefore, modern taxonomy must carefully integrate multi-scale evidence, encompassing micromorphology, anatomy, and geography, within a robust macro-molecular phylogenetic framework.

## 4. Genomics and Karyotype Evolution

*Carex* represents one of the most species-rich genera in the plant kingdom. Despite the formidable challenges posed by holocentric chromosomes, recent innovations in sequencing technologies, spanning karyotype analysis to chromosome-scale genome assembly, are systematically unveiling the genus’s complex genetic background. These advancements are crucial for elucidating the genomic mechanisms driving its rapid lineage diversification and ecological adaptation.

### 4.1. Chromosome, Genome Size, and Karyotype Evolution

The most prominent feature of *Carex* cytogenetics is the tremendous variation in chromosome numbers. Extensive cytological surveys across hundreds of species have revealed a continuous aneuploid series, with haploid numbers ranging from *n* = 13 to *n* = 56 and diploid counts spanning from 2*n* = 18 to 2*n* = 108 [22,23].

This extensive variation stems directly from the unique holocentric structure characteristic of the Cyperaceae. Unlike monocentric organisms, this diffuse centromere organization allows for the stable inheritance of chromosome fission (agmatoploidy) and fusion (symploidy) while avoiding the common loss of genetic material [24]. Although polyploidy events may have occurred during early evolution, holocentric-driven chromosomal rearrangements are widely considered the primary source of this extraordinary variation.

Within the phylogenetic framework of the major *Carex* clades (*Siderostictae*, *Caricoid*, *Vignea*, and Core *Carex*) [25], chromosome evolution operates as a significantly nonuniform process. Evolutionary rates differ vastly among lineages; for instance, the North American section Ovales within the *Vignea* clade exhibits an extremely high rate of chromosome fission [26]. Furthermore, analytical models demonstrate that the evolution of chromosome numbers is not governed by mere random drift. Instead, it is subject to strict selective constraints, tending to converge toward clade-specific stationary distributions.

Genome size is a crucial dimension for understanding karyotype evolution, but its relationship with chromosome number is highly intricate. Empirical measurements across 157 taxa reveal that *1C* values vary significantly, ranging from 0.24 pg in *C. secalina* to 1.64 pg in *C. cuspidata* [27]. Furthermore, among non-polyploids, a strong negative correlation exists between genome size and chromosome number. This pattern supports the hypothesis that fission and fusion events lead to an increased chromosome number accompanied by a decrease in average DNA content per chromosome. However, at deeper evolutionary scales, such as within the *Vignea* clade, the evolution of chromosome number and genome size appears to be completely decoupled [28]. This decoupling suggests that the independent proliferation and removal of long terminal repeat (LTR) retrotransposons may be the specific molecular mechanism driving fluctuations in genome size, a process distinct from karyotype changes driven by chromosome fission or fusion.

Although specific mechanisms remain controversial, drastic karyotypic changes are widely recognized as a core driver of species diversity in *Carex*. Macroevolutionary analyses confirm that karyotypic instability, resulting from a holocentric structure, serves as a key engine for the genus’s evolutionary radiation by promoting reproductive isolation [29]. Notably, this promoting effect is not a simple linear relationship. Lineage diversification rates peak at intermediate recombination rates (chromosome number 2*n* ≈ 60), while rates are lower at extremely high or low chromosome numbers. This indicates the existence of an optimal window for the impact of karyotype evolution on speciation.

### 4.2. Nuclear Genome Assembly and Repetitive Sequence Characteristics

Although *Carex* genomes are relatively small, their holocentric nature and high repetitive sequence content have historically hindered assembly efforts. In recent years, however, the combination of PacBio/Nanopore long-read sequencing and Hi-C technology has catalyzed major breakthroughs in this field. For instance, the first chromosome-scale genomes were successfully assembled for *C. cristatella* (301.6 Mb, anchored to 35 chromosomes) and *C. scoparia* (298.0 Mb) [30]. This progress quickly expanded to geographically distinct taxa, including Qinghai–Tibet Plateau species, resulting in assemblies for the inferred tetraploid *C. parvula* (783.49 Mb) and the inferred triploid *C. kokanica* (673.40 Mb) [31]. The repository of *Carex* genomic resources continues to grow rapidly, now encompassing a draft genome for *C. pumila* (~0.346 Gb; 23,402 predicted protein-coding genes) [32] and a high-quality assembly for *C. breviculmis* (469.01 Mb; 37,372 annotated genes) [33].

Repetitive sequence analysis reveals a high proportion of repetitive elements in *Carex* genomes (e.g., 52.0% in *C. breviculmis* and 52.5% in *C. parvula*) [31,33], with recent bursts of LTR retrotransposons acting as the primary driver of genome expansion. Uniquely, the genomes of *C. cristatella* and *C. scoparia* contain a higher proportion of DNA transposons than retrotransposons, a phenomenon that is relatively rare among plants [30].

In-depth comparative genomic analyses have revealed drastic structural variations resulting from holocentricity. For instance, despite the availability of chromosome-level assemblies, large-scale conserved syntenic blocks are almost undetectable between *Carex* and *Poaceae* species such as rice. This profound lack of synteny indicates that frequent fission and fusion events have extensively reshaped the genomic landscape. Furthermore, the absence of typical tandem repetitive centromeric sequences within these high-quality assemblies strongly corroborates the diffuse nature of their centromeres. In terms of functional evolution, high-altitude species from the Qinghai–Tibet Plateau exhibit a significant expansion of stress-resistance gene families, particularly heat shock proteins [31]. This expansion highlights the critical role of genomic plasticity in facilitating adaptation to extreme environments. Ultimately, these expanding genomic resources lay a crucial foundation for deeply elucidating the molecular mechanisms of holocentricity and the broader adaptive evolution of this genus.

### 4.3. Structural Variation in Organelle Genomes

The evolution of organelle genomes offers complementary insights into understanding the phylogeny of the genus *Carex* while exhibiting unique patterns of variation in its own right.

Regarding chloroplast genomes, in contrast to the highly conserved structure characteristic of most angiosperms, those within *Carex* have undergone significant rearrangement and expansion. Comparative analyses of multiple *Carex* species (including *C. breviculmis*, *C. lithophila*, *C. littledalei*, and *C. siderosticta*) reveal that their genome lengths (181–213 kb) are significantly larger than the typical angiosperm average of approximately 150 to 160 kb [34]. This profound size increase is primarily attributed to the dramatic expansion of the inverted repeat (IR) regions, which, for instance, reach up to 51,303 bp in *C. breviculmis*. Similar characteristics of enlarged genomes are consistently observed across the genus, as demonstrated in *C. agglomerata* (184 kb) and *C. laevissima* (188 kb) [35,36]. Furthermore, breakpoint analyses indicate that these sites of variation overlap highly with long repetitive sequences, strongly suggesting that illegitimate recombination acts as the primary molecular mechanism driving these drastic structural changes.

Mitochondrial genomes within the genus exhibit even greater complexity and instability. For instance, the first completely assembled *Carex* mitochondrial genome (*C. breviculmis*) features a massive circular master conformation reaching 1,414,795 bp in length [37]. Abundant internal repetitive sequences within this genome mediate frequent homologous recombination, allowing it to dynamically exist in multiple alternative conformations. Similarly, recent sequencing efforts demonstrate that the *C. pseudochinensis* mitochondrial genome also approaches 1 Mb (965,836 bp) in size [38]. Collectively, these findings confirm that dramatic genome expansion and structural rearrangement, fundamentally driven by repetitive sequences, are core features of organelle evolution in *Carex*.

### 4.4. Functional Genomics and Molecular Marker Development

Genome and transcriptome sequencing have laid a solid foundation for characterizing functional genes and developing molecular markers within the genus *Carex*. For example, the application of PacBio SMRT technology has enabled the construction of a high-quality, full-length transcriptome for *C. breviculmis*, generating over 60,000 high-confidence transcripts with an average length of 2302 bp. This comprehensive dataset facilitated the unprecedented identification of 3588 alternative splicing events and 1273 long non-coding RNAs (lncRNAs) in this species. Consequently, these molecular resources provide a critical reference for elucidating complex stress-resistance mechanisms, such as shade tolerance [5].

At the genomic level, whole-genome duplication (WGD) events have emerged as a significant driving force for adaptive evolution within the genus *Carex*. Synonymous substitution rate (*K*_s_) distribution analyses robustly confirm that the genus experienced a recent, lineage-specific WGD event. In this evolutionary context, the enhanced environmental adaptability of *C. breviculmis* is largely attributed to the rapid expansion and positive selection of tandemly repeated genes involved in sugar, amino acid, and phenylpropanoid biosynthesis pathways. Similarly, specific gene expression responses to high-altitude cold stress have been well-characterized in species including *C. parvula* and *C. kokanica*, further highlighting the critical role of duplicated genes in facilitating adaptation to extreme environments [31].

Omics technologies have significantly accelerated the development and application of molecular markers within the genus. For instance, a robust set of 13 polymorphic SSR markers effectively distinguishes between two morphologically cryptic subspecies of *C. curvula* (subsp. *curvula* and subsp. *rosae*). Molecular evidence reveals that these subspecies represent distinct gene pools, primarily shaped by profound ecological isolation along elevational gradients [39]. Furthermore, emerging genomic resources, such as the *C. pumila* genome, have facilitated the rapid validation of 30 highly polymorphic SSR markers, exhibiting a substantial mean polymorphism information content of 0.660 [32]. In the context of species delimitation, EST-SSRs have proven invaluable for tracing complex gene flow between the extremophyte *C. angustisquama* and its close relatives. Analytical results demonstrate that while natural *F*_1_ hybridization occurs, subsequent gene introgression via backcrossing is severely impeded. This dynamic confirms that species cohesion is effectively maintained through microhabitat isolation and strict reproductive barriers [40].

## 5. Reproductive Biology and Life History Strategies

As a dominant taxon across demanding ecosystems, particularly northern temperate and Arctic wetlands, *Carex* demonstrates highly diverse life history strategies and remarkable environmental adaptability. Its reproductive and developmental frameworks encompass unique gamete development mechanisms, sophisticated regulation of seed dormancy, and highly efficient modes of clonal propagation.

### 5.1. Gamete Development: Unique “Pseudomonad” and Sex Differentiation

Species within the family Cyperaceae display a highly specific “pseudomonad” pollen development pattern during sexual reproduction [41]. Following meiosis, out of the four resulting microspore nuclei, only one develops into a functional pollen grain, while the remaining three abort. Cytological evidence from *Carex* ciliato-marginata reveals that during the tetrad stage, only the functional nucleus undergoes DNA replication. Conversely, the abortive nuclei possess half the DNA content and present as unreplicated chromosomes [42]. Investigations into *C. blanda* further demonstrate that this process is strictly regulated by the cytoskeleton. Specifically, microtubules and microfilaments guide the functional nucleus to the abaxial end, positioning it adjacent to the tapetum responsible for nutrient supply. Simultaneously, these cytoskeletal elements squeeze the abortive nuclei to the adaxial tip, ultimately completing a highly unequal cytokinesis [43]. This extreme developmental asymmetry is widely regarded as an evolutionary adaptation designed to ensure the concentrated supply of resources to the male gametophyte.

Regarding sex differentiation, *Carex* species are predominantly monoecious and protogynous, serving as a critical evolutionary strategy to avoid self-pollination. While environmental factors, including light, water, and nutrients, significantly influence sex ratios, their regulatory magnitude is generally modest. For instance, across monoecious *Carex* taxa, elevated nutrient levels and high light intensity tend to promote “femaleness” (an increased proportion of female flowers), a pattern highly consistent with fitness gain theories based on resource acquisition capacity. Unexpectedly, however, high water availability appears to induce “maleness” [44]. Despite these environmentally driven statistical shifts, the overarching necessity to avoid self-pollination remains the primary driver maintaining monoecy. In contrast, dioecious species such as *C. picta* exhibit distinct temporal differences in resource allocation between the sexes. Although total reproductive investment is comparable, the timing diverges significantly due to the inherent energetic costs of different reproductive structures. Specifically, male investment is highly concentrated during the flowering period to produce resource-intensive wind-dispersed pollen, whereas female investment peaks during the fruiting period to support less costly, single-ovulate non-fleshy fruits [45]. This staggered allocation strategy effectively maintains a resource balance between the sexes. Furthermore, controlled experiments involving defoliation and inflorescence removal definitively confirm a trade-off between growth and reproduction, demonstrating direct resource competition between vegetative and reproductive tissues.

### 5.2. Ecological Strategies of Seed Dormancy and Germination

**Figure 2:**
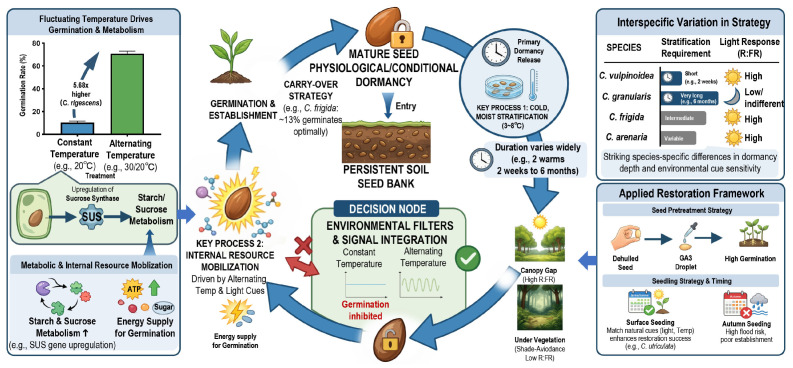
The ecological strategy of *Carex* seed dormancy, germination, and application.

The successful establishment and population recruitment of *Carex* depend largely on sophisticated seed strategies designed to cope with environmental heterogeneity (Figure 2). These strategies typically manifest as complex dormancy patterns and the precise detection of “germination windows”. Seeds of most temperate *Carex* species exhibit strict physiological or conditional dormancy at maturity (e.g., *C. frigida* and *C. ferruginea*, respectively). This mechanism effectively prevents germination during autumn and winter, seasons unfavorable for seedling survival [46,47]. Unlike many other angiosperms, drying after ripening is often ineffective for *Carex* (e.g., *C. divisa*); instead, cold moist stratification has been widely proven to be the most effective method for dormancy release [48,49]. The efficacy and required duration of stratification exhibit high species specificity. For instance, *C. granularis* requires up to six months of stratification, whereas *C. vulpinoidea* requires only two weeks. Furthermore, lower stratification temperatures (3–8 °C) are generally superior to higher temperatures (e.g., 12 °C) for breaking dormancy [46,50].

Once dormancy is released, temperature fluctuations and light signals act synergistically to screen for suitable microenvironments, functioning as an efficient “gap detection” mechanism. Research indicates that *Carex* seeds respond positively to diurnal temperature fluctuations, requiring an average daily amplitude of roughly 10 °C, whereas germination is negligible under constant temperatures (e.g., *C. diandra*). Additionally, the generally high mean temperature threshold required for germination characterizes these species as typical “late spring germinators”, a strategy that effectively avoids early spring cold spells [51,52].

Light constitutes another critical signal regulating germination. The vast majority of *Carex* species are photoblastic, exhibiting significantly higher germination rates in light than in darkness [48,52]. Studies on North American wetland sedges confirm that this photosensitivity is mediated by phytochrome: white and red light promote germination, while far-red light reverses this effect [52]. This dynamic constitutes an efficient “shade-avoidance strategy”. Because vegetation canopies filter out substantial red light, the resulting low red-to-far-red ratio beneath the canopy suppresses phytochrome activity. This inhibition prevents seeds from germinating under dense vegetation and serves as the physiological basis for the formation of persistent soil seed banks.

These environmental signals are intrinsically linked to the seed’s internal physiological state. Recent molecular and physiological studies have elucidated the mechanisms of resource mobilization in response to germination cues. In *C. rigescens*, transcriptomic analysis revealed that alternating temperatures (30/20 °C) increased the germination rate by 5.68-fold compared to constant temperatures. This response is driven by the activation of starch and sucrose metabolic pathways, specifically the upregulation of key genes such as *sucrose synthase* (*SUS*), which provides the energy required for germination [53]. Similarly, for *C. schmidtii*, simulating a natural “drought–rehydration” hydrological rhythm is critical. Maximum germination and seedling vigor were achieved with an initial soil water content of 50% followed by rehydration to 100% on the 14th day. Physiological analysis indicated that this regime maximizes the degradation of stored reserves (starch, soluble proteins, and fats) and the accumulation of soluble sugars [54].

Despite broad similarities in germination strategies within the genus, significant adaptive variation exists. Even among wetland sedges inhabiting similar environments, germination requirements can be strikingly different [46]. These requirements do not correlate significantly with phenology or seed size, although smaller-seeded species generally possess weaker dormancy. Adaptive differentiation is also evident within species across diverse habitats. For instance, *C. pendula* and *C. remota* exhibit distinct responses to stratification temperatures during primary and secondary dormancy, depending on their geographic provenance [55]. Notably, alpine species such as *C. frigida* have evolved a “carry-over” (risk-spreading) mechanism: even under optimal conditions, only approximately 13% of seeds germinate. This strategy ensures that the majority of the seed load remains in the soil bank, providing a crucial buffer against unpredictable environmental fluctuations in high-altitude ecosystems [56].

A profound understanding of these mechanisms is currently being applied to resolve challenges in ecological restoration. Addressing the low natural germination rate of *C. arenaria* (13%), researchers achieved 100% in vitro germination via perigynium removal and GA_3_ treatment. However, hydroponic culture, which yielded 79% germination, produced seedlings with significantly higher vigor and acclimatization survival rates (76%), making it the optimal propagation pathway [57]. For *C. utriculata* and *C. nebrascensis*, autumn seeding poses a risk of flood washout; thus, surface-seeding in early summer, after spring floods recede, is recommended to align with their requirements for light and warm, fluctuating temperatures [58]. Additionally, pre-treating seeds with artificial stratification to accelerate germination is an effective strategy to competitively inhibit invasive species such as *Phalaris arundinacea* [46].

### 5.3. Trade-Offs Between Clonal and Sexual Reproduction

Although *Carex* possesses complex sexual reproductive mechanisms, clonal growth frequently dominates population maintenance and expansion. As typical modular organisms, *Carex* species achieve efficient clonal propagation primarily through the development of rhizomes or tussock formations.

In wetland habitats subject to periodic disturbance, sexual and clonal reproduction exhibit distinct functional differentiation. The dynamics of *C. rugulosa* provide a compelling demonstration of the competitive suppression of sexual recruitment by clonal growth. In mature, high-density clonal populations (590–950 shoots m^−2^), the closed canopy and thick litter layer completely inhibit seedling establishment. Significant germination rates, restoring 17.5% to 39.5% of the seed bank, occur only after ramets are artificially removed or naturally die off due to flooding events. These findings indicate that the primary ecological function of seeds is recovery following disturbance or the colonization of novel patches [59,60]. Beyond reliance on seeds, *Carex* can dynamically adjust its clonal architecture to cope with environmental stress. For example, as sedimentation depth increases, the clonal growth form of *C. brevicuspis* can rapidly shift from a “phalanx” strategy (maintaining local dominance via short rhizomes) to a “guerrilla” strategy (escaping stress via long rhizomes) [4]. This high degree of morphological plasticity serves as a key mechanism for adapting to dynamic wetland environments.

In terms of modular demography, the life history of ramets—the fundamental building blocks of *Carex*—demonstrates profound habitat dependence. Temperate species, such as *C. lacustris*, exhibit rapid population turnover, characterized by ramet lifespans typically lasting only 12 to 24 months and high mortality rates. In contrast, Arctic–alpine species adopt an extreme slow-life strategy to adapt to low temperatures and nutrient poverty. Ramets of *C. bigelowii* can survive for 5 to 7 years, while molecular analyses of the alpine species *C. curvula* have revealed that single clonal genets can persist within the same microhabitat for over 2000 years [61,62]. This extreme clonal persistence effectively minimizes the reliance on high-risk sexual recruitment in harsh environments and represents the ultimate evolutionary outcome of life history trade-offs across distinct climatic zones.

### 5.4. Interspecific Hybridization and Speciation

The developmental strategies of *Carex* are not only reflected in individual life histories but also extend to its dynamic evolutionary processes. Hybridization is extremely common within the genus, profoundly influencing taxonomic patterns and evolutionary trajectories. An early systematic review of North American sedges documented as many as 253 hybrid combinations, noting that the incidence of hybridization in the subgenus *Carex* (78.8% of total reports) was significantly higher than in the subgenus *Vignea* (21.7%). Ecologically, hybridization frequently occurs in disturbed sites or intermediate habitats. Geographic distribution patterns reveal that 84.1% of North American hybrids are distributed in areas affected by Late Pleistocene glaciation. Consequently, the availability of bare ground following glacial retreat, habitat instability, and the overlap of flowering phenology caused by migration convergence are considered key drivers promoting hybridization events [63].

From a cytological perspective, hybrids typically exhibit high sterility due to severe meiotic abnormalities, particularly the frequent occurrence of univalents, multivalents, and laggards. Although *F*_1_ generations are largely sterile, backcrosses can partially restore fertility. Therefore, hybridization has long been regarded as an important evolutionary force driving speciation and generating aneuploid chromosomal variation [63].

However, with the advancement of genomic technologies, increasing evidence suggests that hybridization is not merely an evolutionary “dead end” but a potent catalyst for adaptive evolution. For instance, genotype–environment association analyses of *C. nova*, *C. nelsonii*, and their hybrid zones in the Rocky Mountains demonstrate that hybrid offspring do not simply display intermediate traits. Instead, these lineages acquire unique genetic combinations through adaptive introgression [64]. Such hybrid individuals are often enriched with adaptive variations that enable them to occupy distinct climatic niches inaccessible to either parent, thereby achieving significant niche expansion. This dynamic indicates that, in the context of rapid climate change, interspecific hybridization may provide the essential genetic raw material for *Carex* to adapt to novel environments.

Furthermore, hybridization serves as a primary driver of taxonomic complexity and the formation of cryptic species within *Carex*. Because hybrid offspring are often morphologically indistinguishable from their parents, traditional classification systems frequently fail to achieve accurate delimitation. By integrating morphological data with molecular phylogenetic evidence, for instance, a new species, *C. lucennoiberica*, was successfully identified and described within *Carex* sect. *Phacocystis*. This discovery confirms that hybridization can effectively blur species boundaries while simultaneously leading to the formation of novel taxa [65]. Similarly, genomic approaches such as ddRAD sequencing have been instrumental in rigorously testing historical hybridization hypotheses, as seen in the study of *C. salina* and *C. ramenskii* [66]. These analyses reveal complex reticulate evolutionary relationships along the North Atlantic coast, demonstrating the indispensability of high-resolution genomic data in resolving taxonomic controversies among closely related, hybridizing species.

Notably, despite the prevalence of hybridization, *Carex* species can maintain their evolutionary independence within specific habitats. Strong habitat selection pressures, including extreme soil acidity and heavy metal stress, have been shown to maintain genetic cohesion and prevent species fusion, even in the presence of frequent interspecific hybridization. A compelling case is found in *C. angustisquama* inhabiting the extreme environments of Japanese solfatara fields. Although natural hybridization frequently occurs with the surrounding *C. sociata*, the intense selective forces of the solfatara environment effectively preserve the species’ genetic integrity [40]. This case clearly demonstrates the dynamic balance between reproductive isolation and natural selection in maintaining species boundaries within complex hybrid zones.

## 6. Ecophysiological Adaptation and Abiotic Stress Response

### 6.1. Strategies for Nutrient and Water Acquisition, Transport, and Utilization

*Carex* species have evolved a complex set of strategies to adapt to habitat heterogeneity, encompassing morphological plasticity, physiological trade-offs, and clonal integration mechanisms.

Habitat nutrient availability directly reshapes biomass allocation patterns. Highly productive species (such as *C. acutiformis* and *C. lasiocarpa*) increase aboveground investment and total nitrogen content to establish a competitive advantage for light. Conversely, low-productivity species (*C. diandra*, *C. rostrata*) increase belowground allocation to enhance nutrient acquisition. Because of their inherently slow growth rates, these species exhibit a “nutrient concentrating effect”, resulting in paradoxically elevated leaf nitrogen concentrations [67]. Significant trade-offs exist in nitrogen use strategies. Highly productive species (*C. acutiformis*) prolong the mean residence time of nitrogen by reducing leaf turnover rates. In contrast, low-productivity species (*C. diandra*) rely on higher nitrogen productivity to maintain survival, despite the risk of nitrogen loss associated with high turnover [68]. Notably, when nitrogen supply increases, nitrogen productivity significantly decreases across all species, indicating that plants respond to resource enrichment through physiological downregulation mechanisms.

When subjected to low phosphorus stress, all species reduce their fresh shoot ratio. Among them, *C. rostrata* exhibits extremely high tissue phosphorus content, demonstrating a strategy of “luxury consumption” in response to nutrient pulses [69]. This physiological regulation coexists with the species-specific utilization of insoluble phosphorus sources. While all tested species can utilize Al-P and glycerophosphate, only *C. acutiformis* can efficiently utilize Fe-P and Ca-P, further enhancing acquisition efficiency by increasing the total root absorption surface area. Conversely, *C. lasiocarpa*, originating from P-limited habitats, specializes in utilizing organic phosphorus [70]. Under phosphorus deficiency, morphological strategies diverge among species. *C. lasiocarpa* exhibits a conservative strategy with fixed morphology, whereas *C. diandra* displays significant plasticity, expanding its foraging range by significantly increasing the root weight ratio and specific root length (SRL) [70].

On a temporal scale, *C. aquatilis* in alpine and arctic habitats demonstrates a precise response to seasonal resources. During the winter–spring transition, when soil temperatures hovered near 0 °C, its roots intercepted 63.4% of the isotopic ^15^N injected into the soil, far exceeding the 7.7% captured by microbes. This high interception efficiency is largely attributed to the competitive window opened by the collapse of soil microbial populations during this period. The plants temporarily store the vast majority of nitrogen in the roots, adopting an “absorb–store–utilize” strategy to support summer growth [71].

On a spatial scale, physiological integration is central to coping with resource patchiness. The sand dune species *C. arenaria* shows a strong tendency for resource sharing in nutrient-poor habitats. Its tracer transport distance and translocation amount (which is 20% higher) are both superior to those of wetland species, and this massive transport does not significantly inhibit the growth of donor ramets [72]. Nutrient translocation is often coupled with water transport. When subjected to reciprocal resource heterogeneity, *C. flacca* can assist ramets in acquiring soil nitrogen through “hydraulic lift”. By achieving a “spatial division of labor” within the clonal system through the morphological specialization of interconnected ramets, the species maximizes its overall benefits [73].

### 6.2. Morphological and Functional Traits in Extreme Environments

The heterogeneity of soil factors, particularly nutrient availability and soil pH, serves as a primary driver shaping morphological variation in *Carex*. Within *C. buekii* populations, soil bioavailable K^+^ content acts as a key factor driving plant miniaturization. Specifically, low potassium directly leads to shortened utricle and beak lengths, indicating that this species possesses high phenotypic plasticity rather than an independent taxonomic status [10]. Similarly, the abundance of *C. pulicaris* is synergistically regulated by soil pH and rainfall, exhibiting a decreasing trend as pH and latitude increase [74]. In the extremely acidic habitats of solfatara fields, *C. angustisquama* exhibits unique adaptation mechanisms. The key abiotic stressor limiting its distribution is extremely low pH (<2.0) rather than Al^3+^ toxicity. This profound acidity restricts distribution primarily by inhibiting root elongation rather than inducing direct mortality. Evolutionary analyses suggest that tolerance to low pH is a derived trait acquired following species divergence, whereas high tolerance to Al^3+^ likely represents a pre-adaptation feature [75].

Water and light availability drive significant differentiation in root and leaf functional traits. Across numerous *Carex* species, root anatomy effectively predicts ecological niches. Species from wet habitats develop extensive aerenchyma, whereas species from dry soils tend to develop thicker sclerenchyma and wider steles [76]. At the leaf level, *C. breviculmis* and *C. heterostachya* demonstrate distinct resource acquisition strategies. The former operates as a slow investment-return plant, tolerating barren and arid conditions through thick cuticles, highly developed bulliform cells, and high water use efficiency. The latter functions as a quick investment-return plant, possessing a larger specific leaf area and highly developed vascular tissues, where SLA serves as a key indicator for assessing its light energy use efficiency [77].

To address physical stresses, including high altitude, mechanical trampling, and hydrological fluctuations, *Carex* maintains survival through fine-tuned structural adjustments and phenological shifts. For instance, *C. ecostata* in the western Himalayas significantly increases plant height and root cortical area, alongside accumulating proline and total free amino acids, with increasing elevation up to 2600 m. Furthermore, its sclerenchyma thickness reaches a maximum at 2200 m, reflecting robust adaptation to extreme cold [78]. When subjected to trampling stress, *C. filispica* shifts toward resource-acquisitive root traits. As trampling intensity increases from 0 to 500 passages, the density of its specialized dauciform roots significantly rises, promoting biomass recovery by enhancing phosphorus acquisition [79]. Furthermore, the phenology of floodplain wetland *Carex* is interactively regulated by hydrothermal factors. The start of the growing season before flooding is primarily driven by air temperature, contributing approximately 76.1%. Conversely, the onset of the post-flooding growing season is dominated by the timing of water recession, which accounts for an 80.1% contribution [80].

### 6.3. Physiological, Biochemical, and Molecular Mechanisms of Abiotic Stress

#### 6.3.1. Salt Stress

**Figure 3:**
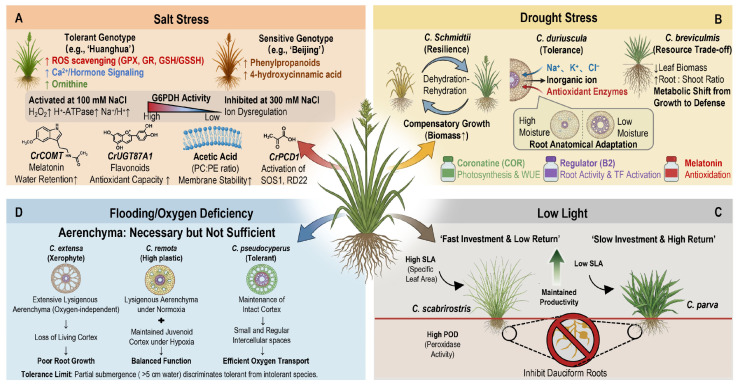
Diverse adaptation strategies of the genus *Carex* to major abiotic stresses. (**A**) Salt-stress responses. (**B**) Drought-stress adaptation. (**C**) Low-light adaptation. (**D**) Flooding or oxygen-deficiency responses.

Research on salt tolerance mechanisms in *Carex* has revealed diverse adaptive strategies across different species and genotypes (Figure 3A). In *Carex moorcroftii*, the glucose-6-phosphate dehydrogenase (G6PDH)-mediated signaling pathway primarily responds to low-concentration salt stress. Under 100 mM NaCl conditions, G6PDH activity increases significantly, inducing the accumulation of the signal molecule H_2_O_2_, which subsequently upregulates the activities of plasma membrane H^+^-ATPase and the Na^+^/H^+^ antiporter. This regulation effectively maintains a high intracellular K^+^/Na^+^ ratio. However, under 300 mM NaCl stress, the activity of this pathway is suppressed, leading to a collapse of ion homeostasis. Exogenous NADPH replenishment experiments indicate that the depletion of intracellular redox power under high salinity is the key limiting factor precipitating the failure of this signaling pathway [81].

Recent investigations into the differential mechanisms between salt-tolerant (‘Huanghua’, ‘Lvping NO.2’) and salt-sensitive (‘Beijing’, ‘Lvping NO.1’) genotypes of *C. rigescens* have substantially enriched the understanding of the salt tolerance network in sedges. Dynamic transcriptome analyses demonstrate that the salt-tolerant genotype activates a markedly higher number of differentially expressed genes under stress (10,752 versus 5764), significantly enhancing the regulation of transcription factors, hormone signaling, and redox networks [82]. Physiological and metabolic evidence corroborates that the tolerant genotype possesses a more robust ROS scavenging capacity (characterized by higher GPX and GR activities, and an elevated GSH/GSSG ratio) and more active Ca^2+^ signaling [83]. Furthermore, this genotype specifically accumulates the proline precursor ornithine and glycine, while promoting trehalose accumulation to maintain osmotic balance through the upregulation of the trehalose-6-phosphate synthase (TPS) gene [82,84]. In contrast, the sensitive genotype relies on the synthesis of phenylpropanoids, including lignin, and the accumulation of 4-hydroxycinnamic acid as supplementary defense measures [83,84].

Building on these transcriptomic insights, the functions of multiple key salt tolerance genes have been successfully elucidated. The caffeic acid O-methyltransferase (*COMT*) gene, which responds rapidly in the tolerant genotype, significantly improves salt tolerance and water retention in transgenic *Arabidopsis* by promoting melatonin synthesis [83,85]. Concurrently, the overexpression of *CrUGT87A1* (a UDP-sugar glycosyltransferase gene) enhances antioxidant capacity through the accumulation of flavonoids [86]. Notably, exogenous acetic acid treatment reveals a novel mechanism of acetic acid-mediated lipid remodeling. This treatment upregulates the content of key phospholipids (phosphatidic acid, PA; phosphatidylcholine, PC; phosphatidylglycerol, PG) and glycolipids (digalactosyl diacylglycerol; monogalactosyl diacylglycerol), increases the PC:PE ratio to maintain membrane fluidity, and reduces toxic free fatty acid content, thereby significantly improving salt tolerance [87]. The gene encoding pyruvate decarboxylase, *CrPDC1*, was subsequently identified as a key downstream effector of this pathway. Its specific upregulation in roots effectively activates stress-responsive genes such as *SOS1* and *RD22* [88].

Furthermore, research on the molecular basis of stress tolerance has expanded to other species within the genus. For example, *ADP*, *TBP*, and *eIF4A* have been screened and verified as the most stable reference genes for RT-qPCR analysis in *C. muskingumensis* under salt and PEG stress. This provides a robust methodological foundation for broad gene expression analysis across the genus [89].

#### 6.3.2. Drought Stress

*Carex* species exhibit diverse and sophisticated drought tolerance strategies (Figure 3B). Some species, including *C. schmidtii*, possess strong resilience; although studies indicate that they are most sensitive to water deficit during the middle growth stage, re-flooding after consecutive drought can restore photosynthetic capacity, as reflected by chlorophyll content and chlorophyll fluorescence-derived parameters such as ΔFv/Fm′, rETRmax, and Ik, while also promoting compensatory growth in leaf area and biomass [90]. The typical drought-tolerant species *C. duriuscula* not only activates the ROS scavenging system (by upregulating CAT, POD, and SOD) and accumulates proline and soluble sugars under stress but, more importantly, utilizes an energetically efficient osmotic adjustment mechanism via inorganic ions. It absorbs large amounts of Na^+^ and Cl^−^ while maintaining stable K^+^ uptake to avoid ion toxicity, thereby rapidly adapting to arid habitats [91]. In *C. breviculmis*, a significant resource trade-off strategy is observed. This species drastically reduces leaf biomass (fresh weight decreased by 61%) to lower transpiration while significantly increasing the root-to-shoot ratio (increased by 223.3%) to maintain water balance. Furthermore, when drought is superimposed with phosphorus deficiency, the leaf N/P ratio and oxidative damage (MDA) peak, indicating that nutritional status further modifies its drought response [92]. Metabolomic analyses reveal that it shifts resources from growth to defense under stress, significantly upregulating key defense metabolites, such as jasmonic acid, pipecolic acid, and neotrehalose (which are negatively correlated with growth indices), and specifically remodeling pathways including aminoacyl-tRNA biosynthesis, the TCA cycle, and starch and sucrose metabolism [93].

The adjustment of root microstructure serves as another important mechanism for *Carex* adaptation to moisture gradients. Research on *C. moorcroftii* demonstrates that as soil moisture decreases, the root transverse section area, thickness of epidermal cells, and area of aerenchyma significantly decrease, effectively shortening the radial water transport distance. Meanwhile, the vascular cylinder diameter, number of vessels, and total vessel area significantly increase, enhancing vertical transport efficiency. A significant complementary relationship exists between the high plasticity of these anatomical structures across different moisture habitats and their low variability (coefficient of variation) within the same habitat, constituting a sophisticated anatomical adaptation to drought stress [94].

In addition to exploring the intrinsic physiological and anatomical adaptation mechanisms of *Carex*, recent research has focused on the application of plant growth regulators to enhance drought resistance. In *C. leucochlora*, the application of coronatine significantly alleviates drought stress, increasing the net photosynthetic rate by 44.8% and water use efficiency by 114.2%, while reducing relative electrolyte leakage by 42.6% [95]. Treatment with the novel regulator B2 (a 2,4-dichloroformamide cyclopropane acid derivative) increases leaf dry weight by 35.0% and root vitality by 100.7% in *C. breviculmis* following drought. The mechanism involves activating AP2/ERF and WRKY transcription factors, significantly upregulating key genes for lignin synthesis (*HCT*, *POD*, *COMT*) and the ABA signaling pathway, and inhibiting starch degradation [96]. Furthermore, the melatonin biosynthesis gene *CrCOMT* is strongly induced by drought in *C. rigescens*, with high expression mainly observed in new tillers and rhizomes. Overexpression of this gene not only increases endogenous melatonin levels and antioxidant enzyme activity in transgenic plants but also significantly reduces MDA content. Exogenous application of melatonin exhibits the same drought-resistance effect [97].

#### 6.3.3. Low Light

Light availability operates as a key limiting factor determining the establishment and maintenance of Carex populations, exerting a particularly significant role during early life history stages (Figure 3C). For the declining wetland species *C. loliacea*, seed germination exhibits a strict light requirement. Specifically, germination is significantly repressed under low-light environments, particularly under “green shade” conditions that simulate vegetation canopies. Although this species can form a persistent soil seed bank and demonstrates seasonal dormancy cycles, breaking dormancy and seedling establishment are highly dependent on light signals (requiring a high R/FR ratio). Furthermore, seedling survival is strongly constrained by light intensity. Increased vegetation density, driven by drainage or forest succession, exacerbates light competition. This dynamic hinders generative reproduction and ultimately drives population decline [98].

For established plants, physiological and morphological adaptation strategies under low-light conditions demonstrate significant interspecific differentiation. Under simulated urban low-light environments (150 μmol·m^−2^·s^−1^), for instance, *C. scabrirostris* adopts a “fast investment–high return” strategy. By significantly increasing peroxidase (POD) activity and specific leaf area (SLA), it maintains higher productivity, demonstrating superior potential for urban understory applications [99]. In contrast, species like *C. parva* exhibit a more sluggish “slow investment–low return” strategy. Despite these divergent approaches, POD activity, SLA, and leaf relative water content consistently emerge as the key factors determining overall photosynthetic capacity. Notably, while low-light conditions completely inhibit the development of specialized dauciform roots in both species, the growth of standard lateral roots remains unhindered. This highlights a critical belowground allocation trade-off: under severe carbon limitation, plants prioritize maintaining basic absorptive functions over investing in high-cost specialized structures.

In-depth molecular investigations further corroborate the complexity of these adaptations. Multi-omics analyses of *C. adrienii* demonstrate that its shade tolerance relies not only on morphological increases in SLA and decreases in the chlorophyll a/b ratio but also involves a profound remodeling of metabolic flux. Shade conditions significantly induce the flavonoid biosynthesis pathway and activate the ABA signal transduction network to coordinate energy allocation between light harvesting and stress defense [100]. Furthermore, certain lineages have evolved unique mechanisms to cope with combined low-light and drought stress. For instance, *C. planostachys* tolerates extreme water potentials as low as −10 MPa under canopy shade. By utilizing the low transpiration demand afforded by the low-light environment, this species successfully occupies dry understory niches where other herbaceous plants struggle to survive [101].

In summary, *Carex* species exhibit diverse adaptive trade-offs under low-light conditions. While some species maintain growth by improving photosynthetic efficiency, expanding SLA, and enhancing antioxidant levels, others, particularly during the regeneration stage, remain highly light-dependent. Consequently, their population dynamics are more susceptible to understory shading and vegetation competition. From the remodeling of molecular pathways to the occupation of specific ecological niches, this species-specific response to low-light stress serves as a critical factor determining the distribution patterns and regeneration capacity of *Carex* in understory habitats.

#### 6.3.4. Flooding/Oxygen Deficiency

In plant responses to flooding stress, the formation of root aerenchyma has long been regarded as central to waterlogging tolerance [102]. However, aerenchyma formation alone cannot fully explain the pronounced differences in adaptive capacity observed among species. For instance, although various alpine *Carex* species successfully induce aerenchyma under hypoxia and survive waterlogged soils, they exhibit significant survival differentiation when subjected to partial submergence (e.g., water levels 5 cm above the soil surface). Under such conditions, species including *C. sempervirens* and even the wetland-adapted *C. davalliana* fail to survive, whereas others, such as *C. nigra*, *C. limosa*, and *C. rostrata*, maintain the highest biomass [103]. This divergence indicates that the mere presence of aerenchyma does not guarantee tolerance to deep submergence. Instead, factors including aerenchyma structural type, overall gas transport efficiency, and potential impediments to gas exchange at the shoot base likely play a much more critical role.

Further anatomical investigations have revealed the structural and physiological basis of differences in waterlogging tolerance. In hypoxic hydroponics, root growth of the xerophytic species *C. extensa* is significantly repressed, whereas *C. remota* and *C. pseudocyperus*, which are adapted to periodic or permanent flooding, exhibit stronger root growth vigor [104]. Anatomical mechanisms demonstrate that this is closely related to root structural strategies. *C. extensa* forms extensive lysigenous aerenchyma regardless of oxygen conditions, leading to a substantial loss of living cortical tissue. In contrast, the most flood-tolerant species, *C. pseudocyperus*, maintains an intact cortex throughout, facilitating oxygen transport through a system of fine and regular intercellular spaces down to the root tip. *C. remota* displays high plasticity, forming lysigenous aerenchyma under aerobic conditions while maintaining a “juvenile growth habit”, characterized by the retention of intact cortical cells, under hypoxia [105]. This structure not only ensures gas transport but also maintains the viability of root cortical cells, thereby safeguarding nutrient uptake in hypoxic environments.

In addition to morphological and anatomical adaptations, energy metabolism and nutrient homeostasis serve as key internal factors determining waterlogging tolerance in *Carex*. Analyses of *C. brevicuspis* reveal that it adopts a “Quiescence Strategy” to cope with deep submergence, suppressing shoot elongation to reduce energy consumption. This strategy relies on the efficient conversion of non-structural carbohydrates (NSC), rapidly converting starch in rhizomes into soluble sugars to provide energy substrates for anaerobic respiration and regrowth after de-submergence [106]. Furthermore, stoichiometric analyses show that this species can maintain strong nitrogen homeostasis under long-term submergence, but phosphorus (P) homeostasis is significantly weakened. This suggests that the root system’s ability to conserve P may represent a potential nutritional bottleneck for adaptation to long-term flooding environments [107].

Overall, waterlogging tolerance in *Carex* depends not only on the presence of aerenchyma but, more importantly, on the type and continuity of the ventilation structure and its ability to balance oxygen supply with the maintenance of root physiological functions (Figure 3D). Concurrently, metabolic flexibility based on NSC reserves and the maintenance of homeostasis for key nutrient elements, specifically N and P, collectively constitute the physiological basis for the survival of flood-tolerant species in complex hydrological environments.

## 7. Community Ecology, Ecosystem Function, and Ecological Restoration

### 7.1. Biological Interactions and Community Assembly

#### 7.1.1. Interaction Mechanisms of Rhizosphere and Phyllosphere Microorganisms

The assembly of *Carex* rhizosphere microbial communities is dually regulated by the soil environment and plant genotype, exhibiting significant spatiotemporal differences. Multi-site investigations of *Carex* arenaria demonstrate that its rhizosphere bacterial community structure is highly similar to that of the bulk soil, where pH, rather than plant genotype, acts as the primary driver. This indicates that its community composition largely depends on the habitat’s indigenous microbial pool [108]. However, recent studies have revealed the ability of *Carex* to enrich specific functional microbial groups. Specifically, comparing between the native species *C. leucochlora* and the non-native *Poa pratensis*, the former not only significantly enhances soil organic carbon (SOC) and enzyme activity but also increases microbial network complexity and vertical connectivity across soil layers (0 to 40 cm). During this process, it specifically recruits key taxa involved in carbon and nitrogen cycling, such as Actinobacteria and Nitrospirae [109].

Regarding fungal interactions, the “non-mycorrhizal” attribute of *Carex* is now understood to be highly context-dependent. In alpine ecosystems on the Qinghai–Tibet Plateau, although *C. capillacea* struggles to independently maintain a mycorrhizal network, it can undergo “passive colonization” when co-existing with host plants (including *Poa annua* and *Medicago sativa*), reaching a colonization rate of 30%. However, only hyphae and vesicles are found inside roots, with no observation of arbuscules responsible for nutrient exchange, confirming its non-functional character [110,111]. Such colonization can even exhibit parasitism under specific contexts. For example, under water deficit, AMF colonization reduces the nitrogen uptake rate and N:P ratio of *C. thunbergii* by downregulating the expression of root ammonium (*CtAMT1.3*) and nitrate (*CtNRT2.5*) transporter genes [112]. In contrast, dark septate endophytes (DSE) functionally substitute for AMF to perform ecological functions in alpine barren habitats. Although they significantly increase host phosphorus content, their growth effects show significant species specificity. DSE significantly promotes biomass accumulation in *C. firma* but does not promote, and may even slightly inhibit the growth of *C. sempervirens* due to carbon drain [113].

Beyond symbiotic fungi, biochemical processes in the rhizosphere microenvironment also shape unique bacterial functional groups. In the dominant wetland species *C. cinerascens*, the radial oxygen loss (ROL) process induces the formation of significant iron plaque on the root surface, a microhabitat that specifically recruits the colonization of iron-oxidizing bacteria. This “root–microbe–mineral” complex structure not only regulates the rhizosphere redox potential but also serves as a barrier against heavy metals, effectively intercepting the translocation of lead (Pb) to the shoots, thereby conferring a survival advantage to the sedge in polluted habitats [114]. Furthermore, in the context of invasion ecology, certain sedges, such as *C. kobomugi*, can displace native species through fundamental community restructuring. It not only exhibits extremely low AMF dependency but also constructs a rhizosphere bacterial network distinct from native species, inhibiting the establishment and restoration of native dune plants through consequent “soil microbial legacy effects” [115].

Investigations of phyllosphere microbiomes predominantly focus on pathogenic fungal diversity. Field surveys have isolated 54 fungal taxa, among which *C. acutiformis* exhibited the highest phyllosphere diversity (38 taxa). These studies clarify specific pathogenic relationships, identifying *Puccinia caricina* var. *caricina* as the main rust pathogen, while *Stagonospora caricinella* causes leaf spot disease in various sedges like *C. riparia*. The activity of these pathogens directly reflects the interaction between host health status and habitat stress [116]. These findings collectively depict a dynamic adaptive landscape of *Carex*, ranging from rhizosphere functional recruitment to microbial symbiotic trade-offs.

#### 7.1.2. Community Assembly Mechanisms and Spatiotemporal Distribution Patterns

In studies of *Carex* community assembly, multi-scale niche differentiation and environmental filtering mechanisms have been confirmed as core drivers maintaining species diversity. At the microhabitat scale, different sedge species in the understory of old-growth deciduous forests show significant differences in their responses to resource gradients. For instance, *C. plantaginea* tends to be distributed in moist habitats with high nitrate availability, whereas *C. backii* and *C. platyphylla* occupy dry habitats and differ in their responses to phosphorus availability [117]. This fine-scale niche differentiation exhibits a strong phylogenetic signal in subarctic fens at the 0.25 m^2^ scale. Tests based on the “limiting similarity hypothesis” demonstrate that closely related species are often significantly negatively correlated (exhibiting mutual exclusion) due to competition for pH and water table depth, while stably co-occurring species mostly originate from distantly related lineages [118]. Beyond competition-driven niche separation, internal self-organization mechanisms within populations also profoundly dictate spatial patterning. For example, populations of *C. stricta* exhibit a highly regular spatial distribution. This remarkable pattern is not a product of underlying environmental heterogeneity but instead emerges from “scale-dependent inhibition”. By inhibiting neighbor growth at close ranges (<15 cm) through localized litter accumulation, the plants spontaneously generate a stable, self-organized population structure with an average spacing of approximately 60 cm [119].

Regarding interspecific interactions, biogeomorphic ecosystem engineering by dominant species plays a critical facilitative role alongside competition. Investigations of the tussock sedge (*C. stricta*) demonstrate that the elevated tussocks, constructed by its roots and litter, create significant microtopographic heterogeneity. The tussock tops provide dry, high-light refuges, enabling numerous flood-intolerant non-wetland plants to coexist within the wetland environment. Quantitative analyses indicate that a single mature tussock (averaging 33 cm in height) can support an average of 7.6 associated plant species. This micro-scale positive interaction significantly offsets the negative effects of environmental filtering [120]. Similarly, plant–soil microbial interactions are important mechanisms for maintaining habitat diversity. In *C. appendiculata* wetlands, increased plant diversity significantly influences the relative abundance of functional bacterial genera (including *Geobacter*) involved in N, S, and C cycling by altering litter inputs. This dynamic reveals a tight above–belowground coupling in community assembly [121].

However, habitat fragmentation and global climate change pose severe challenges to the genetic diversity and distribution patterns of *C. populations*. In *C. davalliana*, fragmented habitats precipitate genetic drift and inbreeding depression, culminating in a 35% reduction in aboveground biomass and a 30% reduction in tiller number [122]. On a large geographic scale, the abundance of *C. pulicaris* is positively correlated with precipitation and soil acidity, indicating that precipitation reduction driven by climate change is a key factor limiting its distribution [74]. Predictive models under future climate scenarios indicate that the narrow endemic species *C. panormitana* faces an approximate 95% risk of losing suitable habitat. Furthermore, even for the widespread species *C. reuteriana*, gene flow between populations may be impeded by climate warming, driving the solidification of genetic structure [123]. These studies emphasize that self-organization mechanisms, biotic facilitation, and macroscopic climate drivers must be comprehensively considered to fully understand the spatiotemporal dynamics of *Carex* communities.

### 7.2. Key Ecosystem Functions

#### 7.2.1. Litter Decomposition and Nutrient Cycling

In wetland ecosystems, the high biomass accumulation of *Carex* is closely related to its unique internal nutrient cycling mechanisms. For instance, the total primary productivity of *C. lacustris* can reach 1173 g·m^−2^ (with 965 g·m^−2^ allocated aboveground), establishing litter as the primary pathway for nutrient return to the soil. However, this process is not a simple linear release but exhibits significant seasonal dynamics. Plants invest substantial annual productivity (approximately 600 g·m^−2^) to construct overwintering green shoots, maintaining a winter standing crop of approximately 178 g·m^−2^. During this phase, mobile elements, specifically N, P, and K, are translocated and stored from senescing leaves. This translocation provides a competitive advantage for growth in the subsequent spring, constituting a highly efficient internal nutrient conservation strategy [124,125].

Litter decomposition rates are dually regulated by habitat conditions and substrate quality, and they do not consistently align with the hypothesis that high-nutrient species inevitably accelerate elemental cycling. For example, the decomposition rate of the highly productive species *C. acutiformis* is significantly slower than that of *C. diandra*, with first-year dry weight losses of 27% and 45%, respectively. Furthermore, *C. acutiformis* almost completely immobilizes N and P over a two-year period. In contrast, K is rapidly released across all species, primarily driven by physical leaching [126]. Mechanistic analyses reveal that decomposition-limiting factors undergo spatiotemporal shifts and are significantly modulated by plant physiological regulation. For instance, while fertilization increases litter nitrogen concentration in *C. rostrata*, it paradoxically decreases it in *C. acutiformis* due to a pronounced “growth dilution effect”, wherein the increase in biomass production exceeds the rate of nutrient uptake. This dilution consequently inhibits the subsequent decomposition of its litter [127].

In addition to substrate quality, the hydrological regime acts as another key external filter regulating decomposition. Investigations of *C. brevicuspis* challenge the traditional paradigm that flooding universally inhibits decomposition. Under flooded conditions, driven by strong physical leaching, the litter mass loss rate and dissolved organic carbon (DOC) release reach peak levels. Surprisingly, this dynamic favors the rapid accumulation of surface SOC more effectively than under drought conditions [128]. Furthermore, the contribution of sedges to the wetland carbon pool exhibits significant organ-specific differences. Although aboveground leaves turn over rapidly, root litter demonstrates extremely strong recalcitrance due to its high lignin content. Molecular evidence indicates that deep peat is predominantly composed of root residues, firmly establishing the dominant role of *Carex* belowground biomass in long-term ecosystem carbon sequestration [129].

In extreme alpine habitats, litter decomposition is accompanied by a characteristic succession of microbial communities. In the Bayinbuluk alpine wetland, physical fragmentation and the release of soluble organic matter, both induced by freeze–thaw cycles, form the material basis for this community succession. During this process, bacterial communities are primarily driven by air temperature and humidity, whereas fungal assemblages are more strongly influenced by substrate quality. As decomposition proceeds, psychrotolerant taxa adapted to cold environments, including *Cryobacterium* and *Mrakia*, are gradually replaced by microbial groups possessing specific metabolic functions, such as *Ilumatobacter* after four months, and *Brevundimonas* and *Paracoccus* after six months. This trajectory indicates that litter decomposition under extreme climatic conditions is fundamentally an ecological succession process, wherein functional microorganisms dynamically adjust to shifting environmental constraints and substrate availability [130].

#### 7.2.2. Carbon Storage and Methane Dynamics

Regarding carbon storage, the unique tussock structure of *Carex* forms a substantial organic carbon pool. Investigations into *C. stricta* demonstrate that its tussocks are primarily composed of roots, rhizomes, and duff, exhibiting an organic matter content as high as 95%. Due to the significantly lower decomposition rate of leaves within the tussock (*k* = 0.26 yr^−1^) compared to the inter-tussock spaces (*k* = 0.39 yr^−1^), these structures exhibit remarkable persistence, with ages exceeding 50 years. They typically contribute 41% to 62% of the total biomass carbon (approximately 843 to 1697 g C m^−2^), representing the second-largest carbon pool after the soil [131]. Furthermore, the integrity of *Carex* tussocks profoundly influences the vertical distribution of the SOC pool. In undisturbed habitats, a distinct SOC accumulation peak occurs in the 11 to 20 cm soil layer. While anthropogenic disturbances (such as mowing and grazing) can increase SOC in the 0 to 10 cm surface layer by improving soil aeration, they disrupt deep-layer accumulation patterns. Soil water content and soil respiration are identified as more critical driving factors for these dynamics than the disturbance itself [132].

In terms of methane (CH_4_) emission dynamics, *Carex* acts as a “conduit” connecting anaerobic soil with the atmosphere, providing both physical transport and substrate supply. Clipping experiments on *C. aquatilis* and *C. rostrata* demonstrate that the aboveground biomass offers negligible resistance to gas transport. CH_4_ is primarily emitted through the lower 15 cm of the plant (leaf sheath bundles) near the water surface rather than through the stomata, sealing the clipped ends can reduce emissions to 65% [133]. Long-term studies confirm that plant-mediated transport accounts for 40% to 70% of the total methane flux. Moreover, green leaf area is strongly positively correlated with CH_4_ flux—an effect particularly pronounced during the senescence period in autumn—indicating that the input of photosynthates to the roots provides essential substrates for methanogens [134].

The rhizosphere of *Carex* is also a crucial site for methane oxidation. Stable isotope tracing confirms that emitted methane is depleted in ^13^C relative to pore water (due to mass-dependent fractionation during passive diffusion), whereas rhizospheric methane is enriched in ^13^C relative to deeper soil layers (as methanotrophs preferentially utilize ^12^C). Mass balance calculations indicate that rhizospheric oxidation can attenuate emissions by 0% to 34% [135]. However, in situ measurements using inhibitors (CH_3_F) reveal that oxidation is highly seasonal; during peak emission periods, its actual mitigative effect is less than 20%. This discrepancy suggests that the high oxidation potential measured by aerobic laboratory incubations (58% to 92%) is often not fully expressed in complex natural habitats [136].

### 7.3. Ecological Restoration and Environmental Governance

#### 7.3.1. Restoration Strategies: Propagule Selection and Hydrological Regulation

In the restoration of *Carex* wetlands, the screening of propagules constitutes the primary step. Natural restoration relying on soil seed banks is often limited, as *Carex* seeds account for a low proportion of the seed bank, exhibit an insufficient germination rate (<20%), and experience a significant loss of viability after six months. However, because their aboveground parts can continuously provide massive amounts of seeds, artificial surface sowing utilizing fresh seeds from the current year, combined with a shallow-water moist environment, serves as an effective replenishment strategy [137,138].

Given the uncertainty of seed germination, seedling transplantation has proven to be a more reliable method. In particular, planting seedlings near the edge of water bodies can yield extremely high survival rates. After surviving the sensitive period in their first growing season, the establishment survival rate of *C. stricta* seedlings can reach 98%, significantly outperforming rhizome transplantation [139]. If rhizomes are used for restoration, the transplantation season is crucial. Because autumn transplantation disrupts the plant’s overwintering mechanism, the survival rate for spring transplantation is significantly higher than for autumn transplantation [140].

Once suitable propagules are established, refined hydrological regulation and micro-topography management serve as the keys to ensuring population maintenance. Macroscopically, *Carex* prefers natural-like water-level fluctuations with high amplitudes, whereas a long-term, stable reservoir-type pattern significantly inhibits biomass accumulation. Hydrology exerts a strong interactive driving effect on phenology. Specifically, temperature dominates the pre-flood growing season, while the timing of water recession determines the post-flood growing season, with populations in high-altitude areas being more sensitive to hydrological shifts [80].

Regulating the duration of summer floods requires a trade-off between restoration goals. For example, a 5.5-month flooding period can maximize the recovery of autumn vegetative biomass, while a 6-month flooding period is beneficial for stimulating sexual reproduction in the following spring [141]. Microscopically, “hummock” micro-topographies constructed by species such as *C. schmidtii* can contribute nearly 89% of the community’s productivity during flooding periods. However, during drought periods, hydrological connectivity must be maintained to prevent a decline in dominance and the invasion of mesophytes [142].

#### 7.3.2. Bioremediation and Eutrophication Control

*Carex* species demonstrate multi-dimensional application potential in wetland restoration, with mechanisms encompassing heavy metal retention and organic matter degradation.

In heavy metal management, *C. pendula* exhibits extremely high tolerance and significant rhizofiltration capacity for lead-containing wastewater. Even in water bodies with a lead concentration of 10 mg·L^−1^, the lead accumulation in its roots can reach approximately 10 times that of the aboveground parts, accumulating up to 1600 mg·kg^−1^ [143]. This characteristic of retaining heavy metals in the belowground parts rather than translocating them to the shoots effectively reduces the migration risk of heavy metals in water bodies, although it inherently limits phytoextraction efficiency. Consequently, restoration projects must be coupled with rigorous biomass harvesting and proper disposal, such as incineration, to prevent secondary pollution.

For organic pollutants with complex compositions, the synergistic effect between *Carex* and rhizosphere microorganisms serves as the key to pollutant degradation. When treating oil sand process-affected water (OSPW) containing toxic naphthenic acid fraction components (NAFCs), wetland systems planted with *C. aquatilis* demonstrate a robust attenuation capacity. These systems significantly reduce the total concentration of NAFCs from an initial 72.1 mg·L^−1^ to 17.1 mg·L^−1^ within 84 days. In contrast, unplanted control groups show no significant change over the same period, only decreasing from 64.5 to 59.0 mg·L^−1^, directly confirming the core role of plants in the system [144]. Furthermore, the bioremediation process alters the chemical fingerprint of the pollutants. High-molecular-weight naphthenic acids decrease, whereas the abundance of compounds with higher oxygen content, including O_3_ and O_4_ types, increases. This shift indicates that the plants promote oxidative degradation, thereby reducing overall toxicity. Nevertheless, under this high-load pollution, the plants exhibit physiological stress symptoms of toxicity, including chlorosis and necrosis, by the end of the exposure period [144].

In terms of eutrophication control and habitat amelioration, the regular harvesting of aboveground biomass, known as paludiculture, is an effective strategy for removing excess nutrients. Nutrient removal potential depends on species productivity rather than habitat preference. High-yielding species, such as *C. acutiformis* and *C. rostrata*, can remove up to one-third of the total nitrogen load in the system. Theoretical models suggest it requires only six to 16 annual harvesting cycles to convert a highly loaded peatland into an oligotrophic state [145]. To ensure lasting effectiveness, the biomass must be completely removed from the wetland to block decomposition and recirculation. Furthermore, the removed high-yielding dry matter can serve as a substitute for fossil fuels, providing additional climate mitigation benefits [145].

Additionally, in the restoration of saline–alkali habitats, *C. aquatilis* adopts a root exclusion strategy to cope with Na^+^ stress. Although its physiological functions face a significant decline when the concentration exceeds 1079 mg·L^−1^, it can still survive in extreme sodium environments of 2354 mg·L^−1^, accumulating approximately three times more Na^+^ in its belowground tissues than in its aboveground parts. This root retention effect helps stabilize the substrate during the initial stage of vegetation reconstruction, thereby creating conditions for the progressive succession of the plant community [146].

## 8. Bioactive Substances and Resource Development

### 8.1. Antioxidant Activity

**Figure 4:**
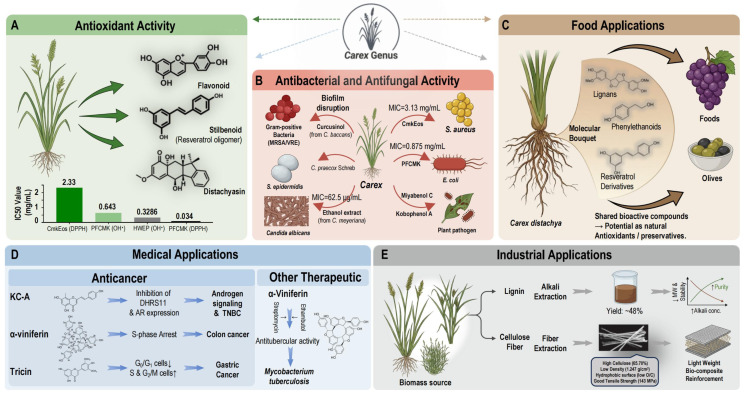
Bioactive compounds and resource-development potential of the *Carex* genus. (**A**) Antioxidant activity and representative phenolic compounds of *Carex* species. (**B**) Antibacterial and antifungal activities of *Carex* extracts or compounds against representative microorganisms. (**C**) Potential food applications of *Carex distachya* related to natural antioxidant and preservative compounds. (**D**) Potential medical applications of *Carex*-derived compounds. (**E**) Industrial utilization of *Carex* biomass for lignin extraction, cellulose fiber production, and bio-composite reinforcement.

*Carex* species, inherently rich in diverse bioactive substances, demonstrate significant application potential in the field of antioxidants [147] (Figure 4A). Phenolic compounds, particularly flavonoids, function as the core contributors to this antioxidant capacity.

The methanol extract derived from the roots of the Mediterranean species *C. distachya* exhibits a robust DPPH radical scavenging ability (*IC*_50_ = 4.2 μg·mL^−1^), which is comparable to commercial antioxidants, including butylated hydroxytoluene (BHT) and ascorbic acid. Furthermore, numerous poly-phenolic components within this extract are also typical constituents of common edible plants, such as grapes and olives, highlighting its potential as a novel and safe food additive [148].

The accumulation of antioxidant substances and their associated mechanisms of action are significantly modulated by environmental habitats. For example, partially submerged *C. acuta* plants accumulate higher concentrations of phenols and flavonoids compared to those grown in moist soil, thereby enhancing their capacity to reduce Fe^3+^ and chelate iron ions. In contrast, the moist soil group performs more effectively in scavenging ABTS radicals, indicating distinct antioxidant strategies driven by habitat variations [149].

Separation and purification processes further amplify antioxidant activity. Purified flavonoids from *C. meyeriana* (PFCMK) exhibit significantly superior scavenging capabilities for DPPH (*IC*_50_ = 0.034 mg·mL^−1^) and hydroxyl radicals (*IC*_50_ = 0.643 mg·mL^−1^) compared to crude extracts, approaching the efficacy of ascorbic acid [150]. Extensive screenings of various *Carex* taxa corroborate these findings. For instance, *Carex divisa* Huds., *Carex melanostachya* M. Bieb. ex Willd., and *Carex stenophylla* Wahlenb. They all contain flavone C- and O-glycosides, while sulfated flavonoids with chemotaxonomic significance are prevalent across most evaluated species [151]. Additionally, the ethyl acetate fractions of species such as *C. petriei* and *C. siderosticta* not only demonstrate high antioxidant activity in DPPH and ORAC assays but also exhibit significant xanthine oxidase (XO) inhibitory activity. This dual functionality reveals a multi-target synergistic mechanism encompassing both the direct scavenging of free radicals and the inhibition of free-radical-generating enzymes [147].

Alongside flavonoids, stilbenes, particularly resveratrol oligomers, constitute another highly scrutinized class of compounds. Chemical isolations from the roots of species including *C. distachya* and *C. capillacea* have yielded compounds such as resveratrol-diglucoside, miyabenol A/C, and α-viniferin. Furthermore, the discovery of a completely novel stilbene tetramer, carexinol A, establishes the genus *Carex* as a natural reservoir of complex and structurally novel poly-phenolic compounds [148,152]. However, comparative analyses indicate that the direct in vitro antioxidant contribution of stilbenes may be secondary to that of flavonoids. For instance, the DPPH scavenging activity of stilbene derivatives, such as pallidol isolated from *C. folliculata* seeds (*IC*_50_ > 1500 μM), is substantially lower than that of flavonoids derived from the same plant (*IC*_50_ values ranging from 57 to 188 μM). Nevertheless, these seed extracts concurrently possess multiple biological activities, including antibacterial properties, highlighting the pharmacological potential of *Carex* seed-derived compounds [153].

Beyond small molecules, macro-molecular polysaccharides also present significant antioxidant potential. Polysaccharides extracted from *C. meyeriana* (CMKP) exhibit robust free radical scavenging capabilities. Specifically, the hot water extract (HWEP) demonstrates optimal efficacy against hydroxyl radicals (*IC*_50_ = 0.3286 mg·mL^−1^), an effect attributed to its high uronic acid content and specific molecular weight distribution [154]. In contrast, the essential oil from the same species exhibits relatively mild antioxidant activity (DPPH *IC*_50_ of 2.33 mg·mL^−1^), primarily because its main constituents are non-phenolic aliphatic compounds, such as palmitic acid [155].

Furthermore, the genus *Carex* serves as an extensive repository for novel antioxidant molecules. Distachyasin, a novel isoprenylated stilbene isolated from *C. distachya* leaves, performs exceptionally well in scavenging superoxide anions and inhibiting TBARS formation [156]. Additionally, two dibenzoxazolone compounds discovered concurrently display potent nitric oxide scavenging ability, along with superoxide anion scavenging capabilities comparable to those of α-tocopherol [157].

In summary, *Carex* species construct a natural, multifaceted antioxidant system driven by the synergistic action of flavonoids, stilbenes, polysaccharides, and other specifically structured molecules. Ultimately, the accumulation and efficacy of these bioactive compounds are co-regulated by both genetic lineage and habitat-induced stress factors.

### 8.2. Antibacterial and Antifungal Activity

*Carex* plants possess an abundance of active antimicrobial components, with modern separation technologies elucidating their pharmacological potential (Figure 4B). Curcusinol, the principal component isolated from the fruits of *C. baccans* (accounting for 3.1% of dry weight), exhibits significant bactericidal and anti-biofilm activities against multidrug-resistant bacteria, including methicillin-resistant *Staphylococcus aureus* (MRSA) and vancomycin-resistant *Enterococcus* (VRE). Furthermore, it functions as an adjuvant to enhance antibiotic sensitivity [158]. In *C. siderosticta*, its isomer curcusionol demonstrates antibacterial activity against *Rhodococcus nicotianae* with a minimum inhibitory concentration (*MIC*) of 12.5 μg mL^−1^, a potency comparable to that of streptomycin. Mechanistic analyses reveal that this compound disrupts cell membrane architecture and inhibits quorum sensing (QS). Additionally, miyabenol C and kobophenol A, isolated from the same species, exhibit inhibitory activity against the phytopathogen *Ralstonia solanacearum* [159]. Evaluations of *C. meyeriana* demonstrate that its essential oil, rich in palmitic and linolenic acids, possesses an *MIC* value of 3.13 mg·mL^−1^ against *S. aureus*, whereas purified flavonoids (PFCMK) exhibit an *MIC* of 0.875 mg·mL^−1^ against *Escherichia coli* [156,160]. Moreover, the ethanol extract of this species strongly inhibits the growth of the pathogenic fungus *Candida albicans* (*MIC* = 62.5 μg mL^−1^) [161].

Broad-spectrum screenings corroborate the universality of antimicrobial activity across the genus *Carex*. Evaluations of 41 European Cyperaceae taxa demonstrate that the ethyl acetate extracts of multiple *Carex* species, including *C. elata*, *C. melanostachya*, *C. oshimensis*, and *C. siderosticta*, consistently exhibit high antimicrobial activity, producing inhibition zones exceeding 15 mm. Notably, *C. oshimensis* displays highly effective inhibition against all tested Gram-positive bacteria. Concurrently, the extract of *C. praecox* produces significant inhibition zones of 18 mm and 14 mm against *Staphylococcus epidermidis* and MRSA, respectively [147,160].

In summary, acting as a natural chemical arsenal, *Carex* species synthesize diverse secondary metabolites, encompassing oligomeric stilbenes, flavonoids, and essential oils. These compounds construct a broad-spectrum and highly efficient inhibitory network against human bacteria, fungi, and agricultural plant pathogens. Consequently, this genus provides a highly valuable source of lead structures for the development of novel natural phytopharmaceuticals targeting multidrug-resistant microbes and plant diseases.

### 8.3. Food Applications

Because they are rich in bioactive polyphenols, including stilbene derivatives, lignans, and flavonoids, species within the genus *Carex* are increasingly regarded as a highly promising source of natural food additives and functional food ingredients (Figure 4C). Comprehensive chemical profiling of *C. distachya* roots has successfully isolated and identified 16 distinct poly-phenolic compounds. These specifically include seven lignans, four phenylethanoids, three resveratrol derivatives, one monolignol, and one secoiridoid glucoside [148]. Many of these components are structurally homologous to the active substances found in common edible plants, such as grapes and olives. This similarity in chemical composition strongly supports the potential application of *C. distachya* root extracts as safe and efficient natural antioxidants within the food industry.

### 8.4. Medical Applications

*Carex* species are rich in stilbenes and flavonoids, which exhibit significant anti-tumor potential [162]. In colon cancer models, luteolin extracted from *C. folliculata* seeds demonstrates inhibitory activity against HCT116 cells (*IC*_50_ = 45 μM) [153]. Stilbene oligomers demonstrate particularly high efficacy. For instance, α-viniferin isolated from *C. folliculata* and *C. gynandra* exhibits *IC*_50_ values ranging from 6 to 32 μM against colon cancer cell lines [163,164]. Concurrently, (-)-hopeaphenol derived from *C. praecox* significantly inhibits Colo 205 and Colo 320 adenocarcinoma cell lines [165].

Specific active components within *Carex* possess significant value in modulating cancer signaling pathways. Kobochromone A (KC-A), derived from *C. kobomugi*, exhibits a potent anti-androgen effect. It inhibits the androgen signaling pathway by suppressing DHRS11 (*IC*_50_ = 0.35 μM) and downregulating the androgen receptor (AR) [165]. Furthermore, in the luminal androgen receptor (LAR) subtype of triple-negative breast cancer (TNBC), KC-A generates a synergistic anti-proliferative effect when combined with the AKT inhibitor capivasertib [166]. Additionally, the flavonoid tricin, isolated from *C. meyeriana*, effectively inhibits the proliferation of gastric cancer SGC-7901 cells. This inhibition is achieved by impeding energy metabolism—specifically reducing lactic acid production, ATP synthesis, and glucose uptake—and inducing cell cycle arrest at the S and G_2_/M phases [167].

In the domains of anti-infection and cardiovascular disease therapeutics, *Carex* species also provide critical lead compounds. Specifically, α-viniferin from the roots of *C. humilis* exhibits potent activity against drug-resistant *Mycobacterium tuberculosis*, demonstrating intracellular *MIC*_50_ values at the micromolar level (2.3 to 4.6 μM) alongside low cytotoxicity [168]. Regarding cardiovascular applications, (-)-hopeaphenol from *C. praecox* functions as an effective inhibitor of angiotensin-converting enzyme (ACE) (*IC*_50_ = 7.7 ± 0.9 μM). It preferentially acts on the N-terminal domain of ACE, thereby providing a novel scaffold for the development of antihypertensive drugs [161] (Figure 4D).

### 8.5. Industrial Applications

In industrial applications, the biomass components of *Carex* plants exhibit substantial potential (Figure 4E).

Investigations into *C. meyeriana* Kunth demonstrate that alkaline extraction methods can achieve lignin yields of up to 48.2%. However, while elevated alkali concentrations enhance purity, they concurrently induce reductions in molecular weight and thermal stability, necessitating a strategic trade-off during processing [169].

Regarding cellulose utilization, the stem fibers of *C. panicea* demonstrate significant advantages as lightweight polymer reinforcements. These fibers possess a high cellulose content (65.7%), low density (1.247 g cm^−3^), and robust thermal stability (stable up to 219.4 °C). Notably, their surface hydrophobicity, indicated by an O/C ratio of 0.17, facilitates effective interfacial bonding with non-polar polymer matrices. Regarding mechanical properties, their moderate crystallinity (56.4%) results in a tensile strength of 143 ± 41 MPa and a Young’s modulus of 5.5 ± 1.86 GPa. Although these values do not represent the absolute maximum for natural fibers, they remain highly competitive, positioning *C. panicea* fibers as a highly promising lightweight bio-reinforcement material [170].

## 9. Conclusions and Future Perspectives

As one of the most widely distributed and species-rich taxa in the Cyperaceae family, the global ecological radiation and broad habitat adaptability of the genus *Carex* arise from its synergistic adaptations across multiple dimensions (Table S1), including genomics, anatomy, and physiological metabolism. The karyotype evolution mechanism of holocentric chromosomes confers upon this genus extremely high genetic plasticity, enabling it to adapt to variable environments through rapid chromosome fission and fusion. Concurrently, the unique “pseudomonad” pollen development and refined seed dormancy regulation strategies facilitate the reproductive success and establishment capacity of its populations. These adaptive traits, from genes to individuals, ultimately translate into significant ecosystem service functions. Whether in maintaining wetland carbon sinks, regulating methane emissions, or bioremediating degraded habitats, *Carex* plants play a critical role. Furthermore, their abundant secondary metabolites, including flavonoids, stilbenes, and specific alkaloids, exhibit substantial potential for application in the pharmaceutical, food, and industrial sectors.

Despite considerable progress in *Carex* research, significant knowledge gaps persist. First, regarding taxonomy, while genomics has provided new perspectives, reticulate evolution and frequent interspecific hybridization continue to obscure the phylogenetic relationships of certain species complexes. Future research must integrate pangenomics with multi-dimensional phenotypic data to construct a more robust taxonomic framework. Second, in the context of global climate change, research on the response mechanisms of this genus, particularly alpine and wetland endemic species, remains insufficient. It is necessary to strengthen long-term in situ observations and molecular mechanism analyses under multi-factor synergistic stress to accurately predict shifts in their distribution patterns. Finally, concerning resource utilization, current research largely focuses on the activity screening of primary extracts, lacking the resolution of biosynthetic pathways for key active molecules and the development of efficient utilization technologies. Future research should aim to bridge the continuum from basic biology to ecological function and resource utilization, thereby providing robust scientific support for biodiversity conservation and the sustainable use of germplasm resources.

## Data Availability

No new data were created or analyzed in this study. Data sharing is not applicable to this article.

## Supplementary Materials

The following supporting information can be downloaded at https://www.mdpi.com/article/10.3390/antiox15070838/s1; Table S1: *Carex* species cited in the manuscript: study place, vegetation/altitude, and covered attribute/function.
